# New treatment for pyridoxine-dependent epilepsy due to ALDH7A1 deficiency: first proof-of-principle of upstream enzyme inhibition in the mouse

**DOI:** 10.1093/braincomms/fcaf397

**Published:** 2025-10-14

**Authors:** Clara D M van Karnebeek, Valérie Gailus-Durner, Udo F Engelke, Claudia Seisenberger, Susan Marschall, Nathalia R V Dragano, Patricia da Silva-Buttkus, Stefanie Leuchtenberger, Helmut Fuchs, Martin Hrabě de Angelis, Ron A Wevers, Curtis R Coughlin, Dirk J Lefeber

**Affiliations:** Emma Center for Personalized Medicine, Departments of Pediatrics and Human Genetics, Amsterdam Gastroenterology Endocrinology Metabolism, Amsterdam UMC, 1105 AZ Amsterdam, The Netherlands; Institute of Experimental Genetics and German Mouse Clinic, Helmholtz Zentrum München, German Research Center for Environmental Health (GmbH), 85764 Neuherberg, Germany; Translational Metabolic Laboratory, Department of Human Genetics, Radboud University Medical Center, 6681 EA Nijmegen, The Netherlands; Institute of Experimental Genetics and German Mouse Clinic, Helmholtz Zentrum München, German Research Center for Environmental Health (GmbH), 85764 Neuherberg, Germany; Institute of Experimental Genetics and German Mouse Clinic, Helmholtz Zentrum München, German Research Center for Environmental Health (GmbH), 85764 Neuherberg, Germany; Institute of Experimental Genetics and German Mouse Clinic, Helmholtz Zentrum München, German Research Center for Environmental Health (GmbH), 85764 Neuherberg, Germany; Experimental Genetics, TUM School of Life Sciences, Technische Universität München, 85354 Freising, Germany; Institute of Experimental Genetics and German Mouse Clinic, Helmholtz Zentrum München, German Research Center for Environmental Health (GmbH), 85764 Neuherberg, Germany; Institute of Experimental Genetics and German Mouse Clinic, Helmholtz Zentrum München, German Research Center for Environmental Health (GmbH), 85764 Neuherberg, Germany; Institute of Experimental Genetics and German Mouse Clinic, Helmholtz Zentrum München, German Research Center for Environmental Health (GmbH), 85764 Neuherberg, Germany; Institute of Experimental Genetics and German Mouse Clinic, Helmholtz Zentrum München, German Research Center for Environmental Health (GmbH), 85764 Neuherberg, Germany; Experimental Genetics, TUM School of Life Sciences, Technische Universität München, 85354 Freising, Germany; German Center for Diabetes Research (DZD), 85764 Neuherberg, Germany; Translational Metabolic Laboratory, Department of Human Genetics, Radboud University Medical Center, 6681 EA Nijmegen, The Netherlands; Department of Pediatrics, Section of Genetics and Metabolism, University of Colorado Anschutz Medical Campus, Aurora, CO 80045, USA; Translational Metabolic Laboratory, Department of Human Genetics, Radboud University Medical Center, 6681 EA Nijmegen, The Netherlands; Department of Neurology, Donders Institute for Brain, Cognition and Behaviour, Radboud University Medical Center, 6525 GA Nijmegen, The Netherlands

**Keywords:** metabolic epilepsy, therapy, mouse model, 2-aminoadipic semialdehyde synthase inhibition, lysine biochemistry

## Abstract

Pyridoxine-dependent epilepsy (PDE) due to recessive *ALDH7A1* mutations is characterized by intractable epilepsy that is often unresponsive to antiseizure medications. Irrespective of pyridoxine (vitamin B_6_) supplementation and lysine reduction therapy, patients present severe residual neurocognitive deficits. We evaluated upstream inhibition of 2-aminoadipic semialdehyde synthase (AASS) as a novel therapeutic strategy to reduce the accumulating metabolites (α-aminoadipic semialdehyde, Δ^1^-piperideine-6-carboxylate, pipecolic acid, 6-oxo-pipecolic acid and 2S,6S-/2s,6R-oxopropylpiperidine-2-carboxylic acid) considered neurotoxic. We utilized an existing mouse knockout model of hyperlysinaemia (*Aass*-knockout) and generated a PDE model, a *Aldh7a1* single knockout model via CRISPR/Cas (clustered regularly interspaced short palindromic repeats and CRISPR-associated protein) and generated the double-knockout *Aass/Aldh7a1* mice. Next-generation metabolomics screening was performed to measure all known biomarkers in brain, liver and plasma of wild-type and mutant mice. Metabolomics confirmed the known metabolite markers for *Aldh7a1*-knockout and *Aass* knockout mice in all samples. The potentially neurotoxic metabolites (Δ^1^-piperideine-6-carboxylate, pipecolic acid, 6-oxo-pipecolic acid and 2S,6S-/2s,6R-oxopropylpiperidine-2-carboxylic acid) significantly decreased in double-knockout *Aass/Aldh7a1* mice brain and liver tissues compared to *Aldh7a1-*knockout mice. Plasma analysis revealed a significant reduction of known biomarkers, suggesting a reliable monitoring option in human patients. We demonstrate the first mammalian evidence that AASS inhibition is a viable strategy to rescue abnormal brain metabolism associated with PDE. This may target the intellectual disability and neurologic deficits caused by persistent lysine catabolic-related neurotoxicity despite adequate vitamin B_6_ supplementation.

## Teaser

van Karnebeek *et al*. report that upstream enzyme inhibition is a viable strategy to rescue the abnormal brain metabolism associated with pyridoxine-dependent epilepsy. This has the potential to replace arduous dietary treatments, reduce the accumulation of neurotoxic metabolites and improve the cognitive outcomes for patients with pyridoxine-dependent epilepsy.

## Graphical Abstract

**Graphical Abstract:**
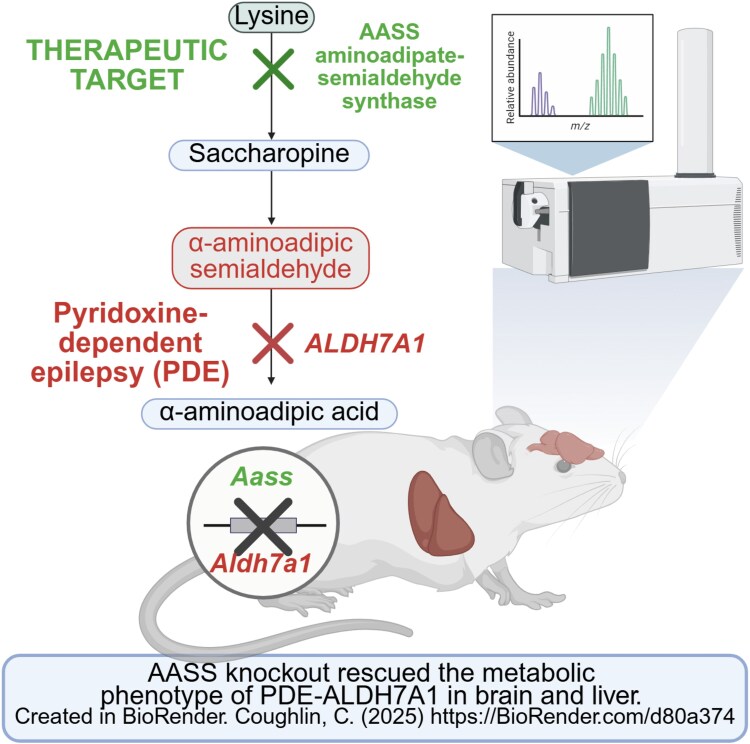

## Introduction

Pyridoxine-dependent epilepsy (PDE-ALDH7A1) is a metabolic encephalopathy characterized by an intractable epilepsy that is often unresponsive to antiseizure medications.^1,2^ Treatment with pharmacologic doses of pyridoxine (vitamin B_6_) results in clinical improvement and long-term seizure control, although intellectual and developmental disability (IDD) persist for the majority of patients.^3^ The degree of cognitive impairment does not correlate with time of seizure onset or severity suggesting a separate disease mechanism for the IDD.^3,4^

**Figure 1:**
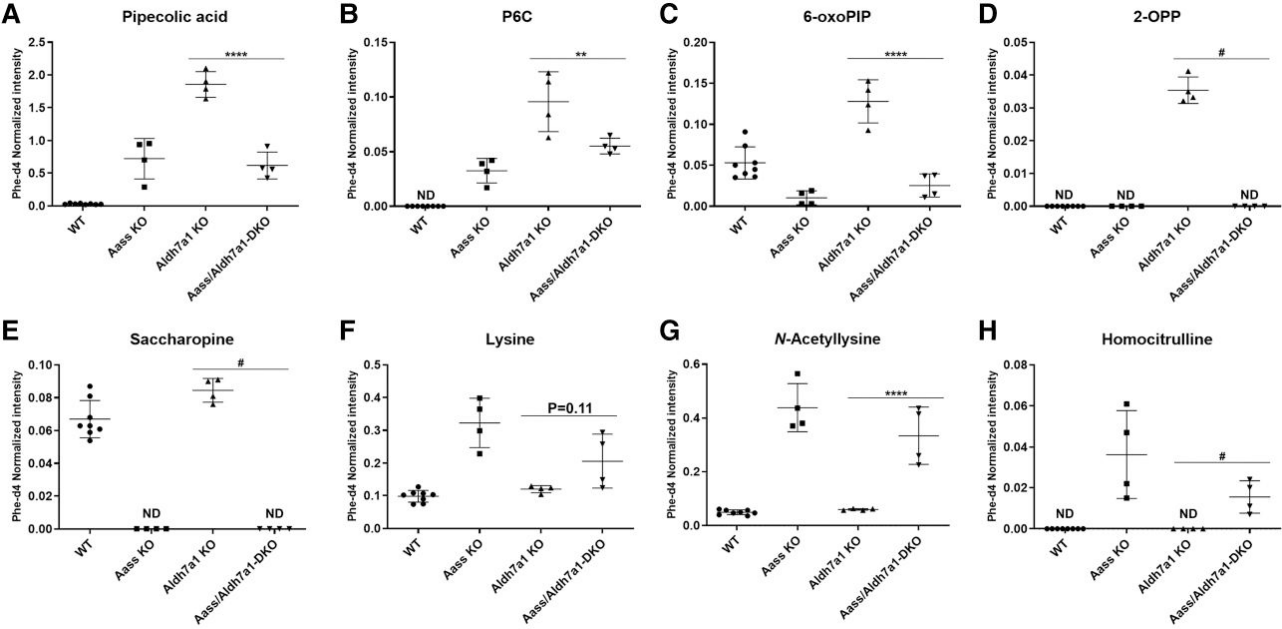
**Brain metabolite profiles** (**A**: pipecolic acid; **B**: P6C; **C**: 6-oxo-PIP; **D**: 2-OPP; **E**: saccharopine; **F**: lysine; **G**: *N*-acetyllysine; **H**: homocitrulline) of mice across various genotypes: WT, KO, *Aldh7a1* KO and *Aass/Aldh7a1* DKO (*n* = 4/group). Data point shows normalized intensities. The error bars represent the mean with standard deviation (SD). The ANOVA yielded a significant *P*-value of <0.0001 for all metabolites. Subsequently, a Tukey’s multiple comparison test was performed to compare the mean of each group with that of every other group. Specifically, the results are presented for the comparison between *Aldh7a1* KO and *Aass/Aldh7a1* double KO (*Aass/Aldh7a1* DKO) groups, indicating a highly significant difference (*P < 0.05, **P < 0.01, ***P < 0.001, ****P < 0.0001). In cases where statistically meaningful analysis was not feasible due to values below the detection limit (ND), ‘#’ was used to indicate obvious differences. Aass = 2-aminoadipic semialdehyde synthase, *Aldh7a1* = aldehyde dehydrogenase 7 family member A1p, P6C = Δ1-piperideine-6-carboxylate, 6-oxo-PIP = 6-oxo-pipecolic acid, 2-OPP = 2S,6S-/2s,6R-oxopropylpiperidine-2-carboxylic acid, ND = not determined.

PDE-ALDH7A1 is caused by the deficiency of α-aminoadipic semialdehyde (α-AASA) dehydrogenase, the enzyme that oxidizes α-AASA to α-aminoadipic acid within the lysine degradation pathway.^5^ This enzyme deficiency results in the chronic accumulation of α-AASA, Δ^1^-piperideine-6-carboxylate (P6C), pipecolic acid and related metabolites (Fig. 1).^6,7^ The most commonly held view is that one or more of the accumulating highly reactive metabolites are neurotoxic and contribute to the IDD phenotype.^8^ Current treatment includes adjunct lysine reduction therapies (LRTs) to pyridoxine in order to decrease α-AASA and related metabolites,^2^ which is associated with significant cognitive improvement compared to treatment with pyridoxine alone.^9^ Pyridoxine and LRTs at any age is associated with mild improvement—but not normalization—in developmental testing, and depended also on age with the most profound effect if initiated in early infancy. Although these results are encouraging, developmental outcomes are still poor. Lysine is an essential amino acid requiring some dietary intake and limiting our ability to eliminate the accumulation of neurotoxic metabolites. Moreover, life-long consumption of medical formula negatively impacts patient quality of life highlighting the need for new treatment approaches.^10^

Pharmacologic inhibition of an upstream enzyme to reduce accumulation of toxic metabolites has been successfully used to treat other inherited metabolic disorders (IMDs), most notably tyrosinaemia type I.^11^ Theoretically, such an approach would reduce the accumulation of neurotoxic metabolites in PDE-ALDH7A1 and provide an alternative to nutritional therapies. The 2-aminoadipic semialdehyde synthase (AASS) enzyme is an intriguing pharmacologic target. AASS is a bifunctional enzyme with two domains: the lysine-ketoglutarate reductase (LKR) that converts lysine to saccharopine and the saccharopine dehydrogenase (SDH) that oxidizes saccharopine to α-AASA.^12^ Patients with mutations in the LKR domain specifically have a benign biochemical disorder of hyperlysinaemia.^13,14^ Furthermore, pre-clinical studies have demonstrated the utility of inhibiting AASS for other disorders of lysine metabolism such as glutaric aciduria type I.^15^

To address the unmet medical need for PDE-ALDH7A1 and develop more effective therapies we evaluated the efficacy of upstream enzyme inhibition in mice. We utilized an existing knockout (KO) mouse model of hyperlysinaemia^15^ and generated a PDE-mouse model by clustered regularly interspaced short palindromic repeats and CRISPR-associated protein (CRISPR/Cas) technology. In our novel double-knockout (DKO) mouse model, we demonstrate that AASS inhibition rescues the abnormal biochemistry associated with ALDH7A1 deficiency. This is the first evidence that inhibition of AASS may be a viable treatment option for patients with PDE-ALDH7A1.

## Materials and methods

### Generation and acquisition of mouse models

The generation of gene knockout cell lines for the Aldh7a1 KO mouse line generation using CRISPR-Cas9 genome editing was performed essentially as described.^16,17^ Guide and primer sequences are displayed in Supplementary Table 2.

At Helmholtz Zentrum Munich the *Aldh7a1* CRISPR/Cas (C57BL6/6NCrl Aldh7a1^em1(IMPC)Hmgu)^ mouse model was generated using the IMPC targeting strategy with CRISPR/Cas technology (https://www.mousephenotype.org/understand/the-data/allele-design/). The *Aldh7a1* mouse model harbours a CRISPR-Cas9-induced deletions of 1906 bp that covers exons 4 and 5 (ENSMUSE00001247473 and ENSMUSE00001218719; https://www.gentar.org).

The *Aass* KO mouse (C57BL/6N-Aass^em1(IMPC)Tcp^) was created as part of the KOMP2-Phase2 project at The Centre for Phenogenomics (Toronto, ON). It was obtained from the Canadian Mouse Mutant Repository. The *Aass* KO mouse model harbours a CRISPR-Cas9-induced deletions, 473-bp + AAACACTTGATGC deletion that covers exons 5 and 6.

*Aass+/−* mice and *Aldh7a1+/−* mice were intercrossed. Double heterozygous mice (*Aass/Aldh7a1+/−*) were intercrossed to generate double KO *Aass/Aldh7a1* mice. Statistical analysis for neonatal genotype ratios was calculated using the Chi-square test.

Mice were fed a standard diet (Altromin 1314) and maintained according to the directive 2010/63/EU, German laws and GMC housing conditions (www.mouseclinic.de), approved by the responsible authority of the district government of Upper Bavaria.

### RNA quality control

For Aldh7a1 as well as Aass RNA was isolated from kidney tissue of homozygous *Aldh7a1* respectively *Aass* knockout animals using a Qiagen RNA isolation Kit and a 1:5 dilution of the cDNA, generated with NEB ProtoScript® II First Strand cDNA Synthesis Kit, was used for PCR with the primers Aldh7a1 Exon 4 and Aldh7a1 Exon 10/11 rev to detect the deletion of exon ENSMUSE00001247473 and ENSMUSE00001218719 and in a minor content deletion of the two expected exons and exon ENSMUSE00001223399 (Supplementary Fig. 1B). For Aass the primer pair Aass ex 3 and Aass ex 9 rev as well as Aass ex2 for and Aass ex9 rev were used to detect the deletion of exon 5 and 6 and to a minor content a deletion of exons 5–7. The mutations were verified by Sanger sequencing.

### Sample collection and analysis of metabolic biomarkers with next-generation metabolomic screening

For metabolomics analysis, samples from the relevant tissues (i.e. where lysine metabolism plays a key role in PDE) including plasma, brain and liver were collected from *Aldh7a1* KO (*n* = 4, 3 m/1f), *Aass KO* (*n* = 4, 4m/0f), *Aass/Aldh7a1* DKO (*n* = 4, 1m/3f) and wild-type (WT) (*n* = 8, 6m/2f and 5m/3f for plasma respectively) adult mice of both sexes.

A comprehensive description of sample collection and preparation and the measurement method is provided in the Supplementary material under ‘Analysis of metabolic biomarkers with NGMS’.

Plasma and tissue samples were analysed using UHPLC-QTOF-MS. Data acquisition was performed in both positive and negative ionization modes. For tissue analyses, feature intensities were normalized by the intensity of Phe_d5. For plasma, intensities were normalized to the mean QC intensity of the respective run. Metabolite identity was assigned based on accurate feature mass and retention time compared to reference compounds, with a mass accuracy deviation of less than 5 ppm and a relative retention time difference of less than 10% from reference compound measurements. Additional details for the metabolites are provided in Supplementary Table 3.

### Statistical analysis

Statistical analysis of biomarkers in plasma, brain and liver samples from WT, KO and double KO mice was achieved using one-way analysis of variance (ANOVA), followed by post hoc Tukey tests for genotype comparisons. Significant effects compared to the WT genotype group are denoted in tables as follows: *P < 0.05, **P < 0.01, ***P < 0.001, ****P < 0.0001. In cases where statistically meaningful analysis was not feasible due to values below the detection limit (ND), ‘#’ was used to indicate obvious differences.

## Results

### Generation of *Aass/Aldh7a1* double-knockout mice

For this study, using the IMPC targeting strategy with CRISPR/Cas technology, we created the Aldh7a1^em1(IMPC)Hmgu)^ mouse model (*Aldh7a1* KO) and crossed it to -Aass ^em1(IMPC)Tcp^ to establish an *Aass/Aldh7a1* DKO mouse model (*Aass/Aldh7a1* DKO) (Supplementary Fig. 1A) To generate an experimental cohort, we intercrossed *Aass/Aldh7a1* double heterozygous mice. All mice were kept on a standard diet. The genotype distribution in the progeny (683 pups) was according to the expected Mendelian ratio (Supplementary Table 1). All different genotypes were present and viable at weaning age. We collected organs and plasma from early adult mice of all groups to study key diagnostic markers for PDE.

### Knockout of AASS in PDE mice results in normalization of PDE biomarkers in brain, liver and plasma

#### Brain metabolite analysis (Fig. 1)

*Aldh7a1* KO mice: Given the neurological phenotype associated with PDE-ALDH7A1 deficiency, we first analysed brain metabolite levels to confirm the effect of *Aldh7a1* knockout. In the *Aldh7a1* KO mice, the pipecolic acid levels were approximately 64 times higher than in the WT mice, and the P6C levels were significantly elevated compared to undetectable levels in WT mice (Fig. 1A and B). Recently discovered PDE-ALDH7A1 biomarkers, 6-oxo-pipecolic acid (6-oxo-PIP) and 2S,6S-/2s,6R-oxopropylpiperidine-2-carboxylic acid (2-OPP), were also significantly elevated, with 2-OPP showing the highest increase and being detectable only in the *Aldh7a1* KO mice (Fig. 1C and D). The 6-oxo-PIP levels were elevated to a lesser extent, approximately two times higher compared to WT mice. These increases in metabolites are fully consistent with PDE-ALDH7A1 deficiency.

*Aass* KO mice: We next investigated metabolite levels in *Aass-*KO mice harbouring a mutant allele with a loss of function of both LKR and SDH activities, which is biochemically equivalent to complete inhibition of LKR.^12^ The mice showed a complete absence of saccharopine, indicating a full knockout of the enzyme AASS-LKR (Fig. 1E). Compared to the WT mice, the high fold change (FC) in pipecolic acid (FC = 28), lysine (FC = 4), *N*-acetyllysine (FC = 8) and homocitrulline (FC = 22) further confirmed the defect of the AASS-LKR enzyme. In the *Aass-*KO mice, an increase in P6C levels was also observed compared to the non-detectable levels in the WT mice. However, the increase was less pronounced compared to the *Aldh7a1* KO mice while levels of 6-oxo-PIP and 2-OPP were normal (Fig. 1C and D).

As expected, lysine and *N*-acetyllysine levels were elevated in the *Aass* KO mice, with FCs of 3 and 10, respectively (Fig. 1F and G). Homocitrulline was also clearly present in the *Aass* KO mice, whereas it was undetectable in the WT mice (Fig. 1H). These increases in metabolites are fully consistent with hyperlysinaemia.

*Aass/Aldh7a1* DKO: To test our hypothesis of a normalization of the PDE biochemical phenotype due to *Aass* inhibition, we analysed the effect of the DKO (*Aass/Aldh7a1*) on PDE biomarkers. Like the *Aass* KO mice, saccharopine was undetectable in the DKO mice, while it was always present in the control animals (Fig. 1E), thereby confirming the DKO model. The excretion of PDE biomarkers P6C and pipecolic acid returned to levels similar to those of *Aass* KO mice, decreasing by a factor of ∼2 compared to *Aldh7a1* KO mice (Fig. 1A and B). The levels of PDE biomarkers 6-oxo-PIP and 2-OPP completely normalized in the *Aass/Aldh7a1* DKO mice, comparable to WT mice (Fig. 1C and D). These results indicate that inhibition of AASS expression restores the levels of all relevant biomarkers for PDE-ALDH7A1 in brain tissue, including the suspected neurotoxic metabolites.

#### Liver metabolite analysis

**Figure 2:**
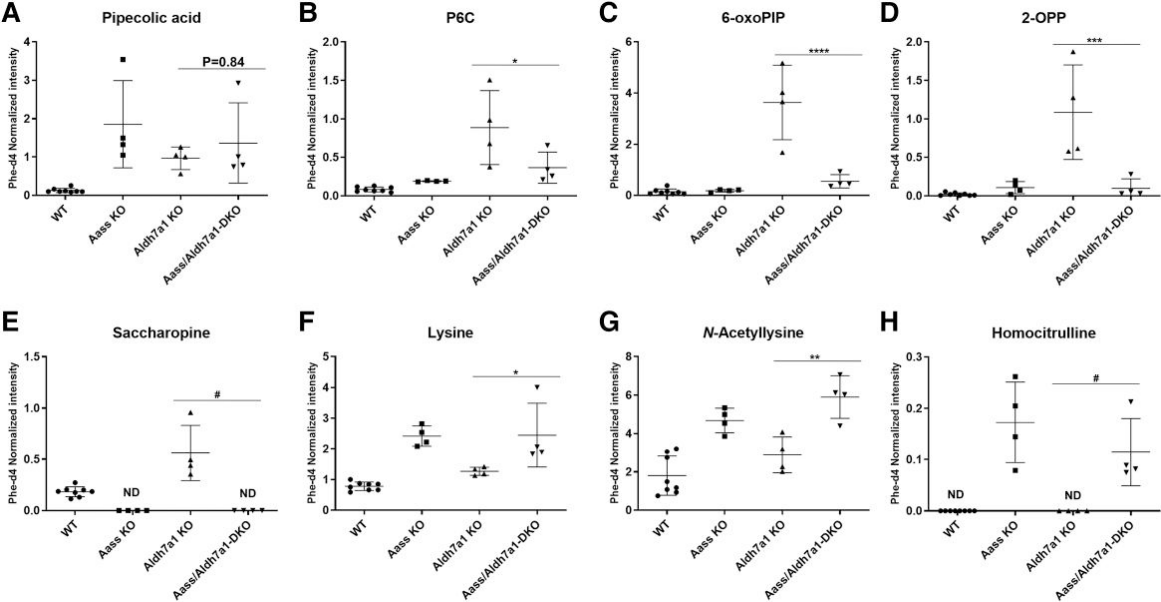
**Liver metabolite profiles** (A: pipecolic acid; B: P6C; C: 6-oxo-PIP; D: 2-OPP; E: saccharopine; F: lysine; G: *N*-acetyllysine; H: homocitrulline) of mice across various genotypes: WT, KO, *Aldh7a1* KO and *Aass/Aldh7a1* DKO (*n* = 4/group). Data point shows normalized intensities. The error bars represent the mean with standard deviation (SD). The ANOVA yielded a significant *P*-value of <0.0001 for all metabolites. Subsequently, a Tukey’s multiple comparison test was performed to compare the mean of each group with that of every other group. Specifically, the results are presented for the comparison between *Aldh7a1* KO and *Aass/Aldh7a1* double KO (*Aass/Aldh7a1 DKO*) groups, indicating a highly significant difference (*P < 0.05, **P < 0.01, ***P < 0.001, ****P < 0.0001). In cases where statistically meaningful analysis was not feasible due to values below the detection limit (ND), ‘#’ was used to indicate obvious differences. Aass = 2-aminoadipic semialdehyde synthase, *Aldh7a1* = aldehyde dehydrogenase 7 family member A1p, P6C = Δ1-piperideine-6-carboxylate, 6-oxo-PIP = 6-oxo-pipecolic acid, 2-OPP = 2S,6S-/2s,6R-oxopropylpiperidine-2-carboxylic acid, ND = not determined.

The liver shows a similar metabolic pattern as observed in brain across the three genotypes of mice, including the absence of saccharopine in *Aass* KO and *Aass/Aldh7a1* DKO mice, indicating complete inhibition of the AASS enzyme (Fig. 2E). Liver levels of pipecolic acid and P6C in the *Aldh7a1* KO mice were elevated compared to WT mice, however less pronounced than in brain, suggesting that the pipecolic acid pathway is the primary lysine metabolic route in the brain (Fig. 2A and B). In contrast, saccharopine levels were higher in the liver compared to the brain (3-fold increase versus normal), indicating that the saccharopine pathway is the primary lysine metabolic route in the liver. As expected, in the liver of *Aass/Aldh7a1* DKO mice, pipecolic acid levels were also increased, along with other hyperlysinaemia biomarkers lysine, *N*-acetyllysine and homocitrulline (Fig. 2F and G and H). Again, the combined data indicate that AASS inhibition results in near complete restoration of the main PDE biomarkers, 6-oxo-PIP and 2-OPP (Fig. 2C and D).

#### Plasma metabolite analysis

**Figure 3:**
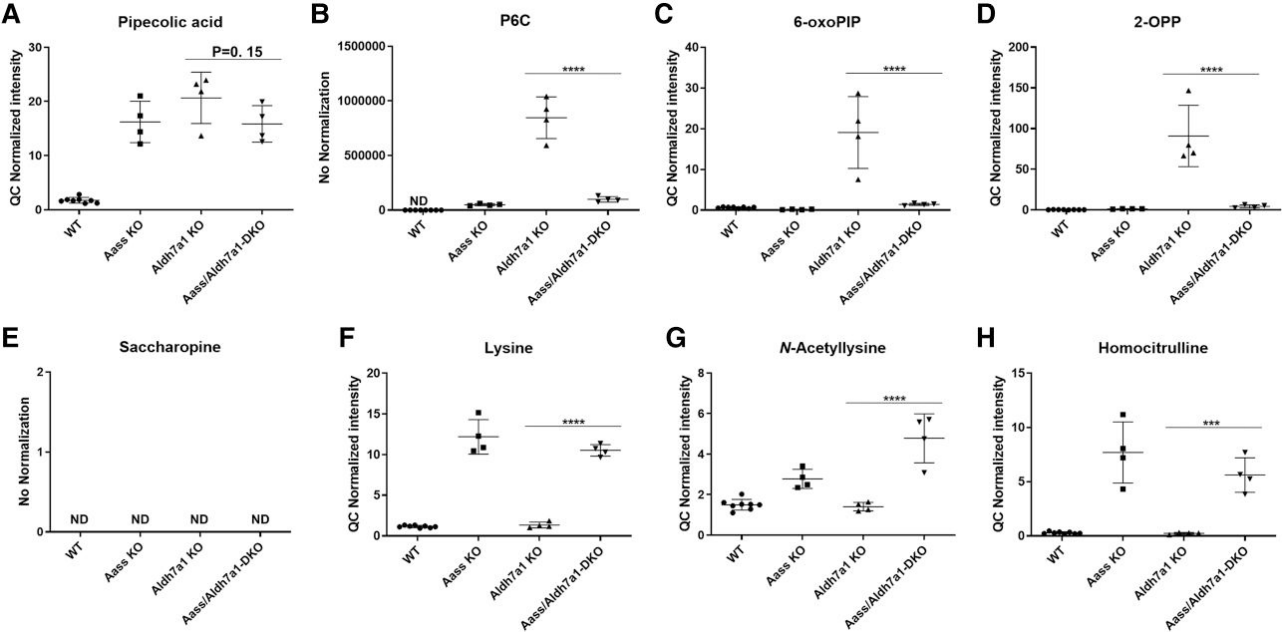
**Plasma metabolite profiles** (**A**: pipecolic acid; **B**: P6C; **C**: 6-oxo-PIP; **D**: 2-OPP; **E**: saccharopine; **F**: lysine; **G**: *N*-acetyllysine; **H**: homocitrulline) of mice across various genotypes: WT, KO, *Aldh7a1* KO and *Aass/Aldh7a1* DKO (*n* = 4/group). Data point shows normalized intensities. The error bars represent the mean with standard deviation (SD). The ANOVA yielded a significant *P*-value of <0.0001 for all metabolites. Subsequently, a Tukey’s multiple comparison test was performed to compare the mean of each group with that of every other group. Specifically, the results are presented for the comparison between *Aldh7a1* KO and *Aass/Aldh7a1* double KO (*Aass/Aldh7a1 DKO*) groups, indicating a highly significant difference (*P < 0.05, **P < 0.01, ***P < 0.001, ****P < 0.0001). In cases where statistically meaningful analysis was not feasible due to values below the detection limit (ND), ‘#’ was used to indicate obvious differences. Aass = 2-aminoadipic semialdehyde synthase, *Aldh7a1* = aldehyde dehydrogenase 7 family member A1p, P6C = Δ1-piperideine-6-carboxylate, 6-oxo-PIP = 6-oxo-pipecolic acid, 2-OPP = 2S,6S-/2s,6R-oxopropylpiperidine-2-carboxylic acid, ND = not determined.

To determine if we can easily monitor the reduction of PDE-ALDH7A1 metabolites in *Aass/Aldh7a1* DKO mice, we also measured plasma metabolites from the mice examined. All PDE-ALDH7A1 and AASS biomarkers were elevated in the *Aldh7a1* KO and *Aass* KO mice, respectively. The only exception was saccharopine, which was not detectable in any of the genotyped mice (Fig. 3E). This contrasts with the brain and liver, where saccharopine was detectable in the WT and *Aldh7a1* KO.

In the *Aass/Aldh7a1* DKO, the levels of pipecolic acid was high for all three disease genotypes, as this is a plasma biomarker for both PDE-ALDH7A1 and AASS diseases (Fig. 3A). The levels of PDE-specific biomarkers P6C, 6-oxo-PIP and 2-OPP strongly reduced in the DKO mice as compared to *Aldh7a1* KO mice, close to the levels seen in WT and *Aass-* KO mice. These results indicate that plasma PDE biomarkers are very well suited to monitor the effect of AASS inhibition in future human trials.

## Discussion

PDE-ALDH7A1 is a readily treatable epilepsy, as pharmacologic doses of B_6_ improve otherwise intractable seizures. Unfortunately, clinical outcomes are still very poor. Even those patients diagnosed and treated antenatally or in the newborn period suffer from developmental delay and neurocognitive impairment.^9^ Treatment with LRTs, in addition to B_6,_ can reduce the risk and severity of IDD,^2^ although life-long nutritional therapies are burdensome for patients and families with inherent compliancy issues. The need for more effective therapies motivated us to investigate a novel approach to the reduction of neurotoxic metabolites.

Using an *Aass/Aldh7a1* DKO mouse, we modelled the impact of upstream enzyme inhibition for the treatment of PDE-ALDH7A1. In both brain and liver, the KO of the enzyme AASS resulted in the normalization (compared to wild-type) of the PDE-ALDH7A1 metabolites 6-oxo-PIP and 2-OPP. It is important to note that, in patients, the combination of B_6_ and LRTs is able to significantly *reduce* the amount of these metabolites although they are not expected to normalize to the level of unaffected patients.^7,18,19^ In the DKO mouse, the levels of P6C and pipecolic acid were similar to those of *Aass* KO mice. These results were expected as pipecolic acid is elevated in patients with hyperlysinaemia,^20^ and, in essence, we exchanged the disorder PDE-ALDH7A1 for hyperlysinaemia.

These results suggest the inhibition of the enzyme AASS has the potential to replace the current treatment regimen. It should be noted that metabolic diets consisting of medical formula are associated with a negative impact on health-related quality of life and an increase in personal and financial burden.^10^ As a result, even if upstream enzyme inhibition was equivocal to current nutritional therapies, it may significantly improve patient and family quality of life. These results suggest that genetic KO (or knock-down) of *Aass* has the potential to be more effective than current therapeutic approaches, especially if administered early in life, ideally in the newborn period.

The enzyme AASS was selected as a target for upstream therapy as patients appear to have a benign biochemical phenotype.^21^ We recognize that long-term follow up of patients with hyperlysinaemia are limited. It remains possible that AASS upstream inhibition could result in ‘trading one disorder for another’, albeit a much less severe condition.^14^ It is also important to note that upstream enzyme inhibition is already standard of care for other IMDs. One such example is tyrosinaemia type I (MIM 276700). Patients with this disorder often present in early childhood with liver and renal dysfunction, severe autonomic crises, and, if untreated, death usually occurs before the second decade of life due to liver failure or hepatocellular carcinoma.^22^ Nitisinone (or NTBC) is ta potent inhibitor of 4-hydroxyphenylpyruvate dioxygenase, an enzyme upstream of the enzymatic defect in tyrosinaemia type I, reducing the accumulation of toxic metabolites, dramatically improving the natural history of this disorder.^11,23^ This treatment is not completely benign as it causes the disorder tyrosinaemia type III with a risk of corneal opacities and other complications. Although not begin, NTBC eliminates the risk for the serious and sometimes even fatal complications of tyrosinaemia type I.^24^

For PDE-ALDH7A1, the clinical effects of our upstream inhibition strategy—i.e. whether biochemical changes align with meaningful correlate with meaningful improvements of epilepsy, cognition and neurodevelopment—require neurobehavioral assessments in mouse, and this is currently ongoing. One limitation of our work is that the number of transgenic mice studied is quite small, albeit sufficient. Another limitation is the reliance on biochemical parameters alone to determine efficacy of AASS inhibition for PDE-ALDH7A1 and other disorders of lysine metabolism such as glutaric aciduria type I.^15^ This is a limitation of the *Aldh7a1* mouse model, which does not have a naive neurologic phenotype.^25^ Of note, current standard of care recommendations for patients with PDE-ALDH7A1 are also based, in large part, on improvement of the same metabolites.^2,18,19,26^ Another limitation is the use of germline KO to model the impact of AASS inhibition. Tissue-specific KO as well as partial enzymatic inhibition (knock-down) and intervention later in development or life (prenatal or postnatal) are currently under study. The same is true for the long-term effect of altered lysine metabolism, such as malnutrition and metabolic side-effects.^30^ Indeed, identifying a viable approach to inhibition of the AASS enzyme for patients with PDE-ALDH7A1 and is a primary aim of our Changing rare disorders of lysine metabolism (CHARLIE) consortium and the focus of future studies using RNA, genomic and pharmacologic technologies in our preclinical PDE-ALDH7A1 disease models including proof-of-principle in patient-derived and CRISPR/Cas9 induced pluripotent stem cells,^27,28^ as well as zebrafish and (dietary manipulated) mouse models. Simultaneously, researchers in the CHARLIE consortium https://www.charlie.science/ partner with patients and families to achieve trial readiness with an international registry in place, with studies on natural history, biomarkers, patients’ experience and outcomes underway.^9,29,30^ We focus on the specific challenges for translation into the clinical arena, which include among others the small numbers inherent to the disease rarity, phenotypic and outcome heterogeneity, differing ages and disease stages, administration route, dosing, safety and long-term effect monitoring.

In conclusion, this study provides the first evidence that inhibition of AASS reduces the neurotoxic metabolites in PDE-ALDH7A1. Upstream inhibition of this enzyme is a promising target to improve the biochemistry, the clinical outcomes and quality of life for affected patients and their families.

## Supplementary Material

fcaf397_Supplementary_Data

## Data Availability

The mouse models used and generated in this study are available either from the International Mouse Phenotyping Consortium (IMPC) or upon request. Aass KO: https://www.mousephenotype.org/data/genes/MGI:1353573#order. Aldh7a1 KO: https://www.mousephenotype.org/data/genes/MGI:108186#order. The metabolomics data presented in this study are available upon request from the corresponding author. No codes were generated or used.
