# Supplementary material for: New treatment for pyridoxine-dependent epilepsy due to ALDH7A1 deficiency: first proof-of-principle of upstream enzyme inhibition in the mouse
Source: Brain Commun. 2025 Oct 14;7(6):fcaf397. doi: 10.1093/braincomms/fcaf397 (PMC12598649; doi:10.1093/braincomms/fcaf397)
Supplement: fcaf397_Supplementary_Data [file fcaf397_supplementary_data.pdf]

Supplementary Materials

Methods: Analysis of Metabolic Biomarkers with NGMS

Sample Collection and Preparation

All mice with genotypes were compared that result from for the purpose appropriate breeding. Single individual animals that obviously deviate from the population, where it can be excluded that this deviation is caused by the mutation and where the inclusion of this animal would systematically confound the experiment, are excluded as an a priori criterion. In general, no data point will be excluded from the analysis, unless there is clear evidence of e.g. technical failure. For the experimental group we did not exclude any data point from the analysis.

For metabolomics analysis, samples from the relevant tissues (ie where lysine metabolism plays a key role in PDE) including plasma, brain, and liver were collected from *Aldh7a1* KO (n=4, 3m/1f)), *Aass KO* (n=4, 4m/0f)), *Aass/Aldh7a1* DKO (n=4, 1m/3f)), and wild-type (WT) (n=8, 6m/2f and 5m/3f for plasma respectively) adult mice of both sexes.

Mice were dissected, and blood samples were collected in Li-heparin-coated tubes, centrifuged at 4500xg for 10 minutes, and plasma aliquots were separated for further analyses. Brain and liver samples were promptly snap-frozen with isopentane in dry liquid nitrogen after collection. Extractions were performed on brain and liver tissues for NGMS analysis.

Approximately 200 mg (+/- 50 mg) of each tissue was weighed. In an Eppendorf tube, 1000 µL of H2O was added. The tissues were then pulverized and centrifuged for 20 minutes at room temperature. The resulting supernatant was used for subsequent sample preparation for NGMS analysis.

Sample preparation was performed as previously described.[30] Briefly, plasma and tissue extract samples were thawed at 4°C and vortexed. A 100 µL aliquot was transferred to a 1.5 mL microcentrifuge tube, followed by the addition of 400 µL of ice-cold methanol/ethanol (50:50 vol/vol) containing 5 internal standards, including L-phenyl-d5-alanine for normalization. The mixture was vortexed for 30 seconds, incubated at 4°C for 20 minutes, and centrifuged at 18,600g for 15 minutes at 4°C. A 350 µL aliquot of the supernatant was transferred to a new tube, dried using a centrifugal vacuum evaporator, reconstituted in 100 µL of water with 0.1%

formic acid, vortexed for 15 seconds, and centrifuged again. A 90 µL aliquot was placed in autosampler vials for analysis at 4°C or stored at –80°C.

NGMS Analysis

Plasma and tissue samples were analyzed using a reversed-phase ultra-high performance liquid chromatography-quadrupole time-of-flight mass spectrometry (UHPLC-QTOF-MS) method. The validation of this platform has been previously described for heparin-anticoagulated plasma.[1] Samples were measured in different batches, each containing mice or patient samples, a performance-check quality control plasma sample (QC), and a solution of internal standards (IS). Both QC and IS solutions were measured at the beginning and end of each analytical batch. Data acquisition was performed in both positive and negative ionization modes. For semi-quantitative tissue analyses, feature intensities were normalized by dividing them by the intensity of the stable isotope labeled internal standard Phe_d5 that was added in every tissue sample. For plasma, feature intensities were normalized to the mean quality control (QC) intensity of the respective run.

Metabolite identity was assigned based on accurate feature mass and retention time in comparison to reference compounds, with a required mass accuracy deviation of less than 5 ppm and a relative retention time difference of less than 10% from reference compound measurements. Additional technical details are provided in Supplemental Table 3.

References

1. Coene KLM, Kluijtmans LAJ, van der Heeft E, *et al.* Next-generation metabolic screening: targeted and untargeted metabolomics for the diagnosis of inborn errors of metabolism in individual patients. *J Inherit Metab Dis* 2018;*41*(3):337-353. doi: 10.1007/s10545-017-0131-6.

Supplementary Tables

Supplementary Table 1: **The genotype distribution in a cross of *Aass*$^{+/-}$/*Aldh7a1*$^{+/-}$ x *Aass*$^{+/-}$/*Aldh7a1*$^{+/-}$ mice**

Observed and expected (in parenthesis) progeny. Expected numbers are calculated based on the total number of pups. According to Chi-Square test the observed genotype distribution does not differ from the expected distribution.

Genotype	*Aldh7a1*$^{+/+}$	*Aldh7a1*$^{+/-}$	*Aldh7a1*$^{-/-}$	Sum
Aass$^{+/+}$	41 (42)	78 (85)	36 (42)	155 (169)
Aass$^{+/-}$	89 (85)	172 (171)	109 (85)	379 (341)
Aass$^{-/-}$	36 (42)	82 (85)	40 (42)	158 (169)
Sum	166 (169)	44 (43)	185 (169)	683

**Supplementary Table 2: Guide sequences used for the CRISPR-Cas9 targeting.**		
Target gene	Exon	Guide sequence
Aass	7 (5’ or + strand)	TGGGGCTCCACATATTCACA
Aass	9 (5’ or + strand)	CTTACTTGTGTGGTAATGCA
Aldh7a1	5' Exon 4	TTCCTGGAGAGGACGTACCT
Aldh7a1	5' Exon 4	GTGGTAGAATCCTTCGAGTG
Aldh7a1	3' Exon 5	TTGTCCCGTGCTTAAGAACG
Aldh7a1	3' Exon 5	GAAACCTCGTTCTTAAGCAC
Primers for PCR amplification and sanger sequencing		
Target gene	Product size	Primer sequence
Aass	wt: 319 bp<br>mut: 561 bp	Aass wtF1: 5’-GATATGCAGACAGGAGAGGTTAACC-3’<br>Aass wtR1: 5’-CAGAGCCAGAACAATAAGAAGACC-3’<br>Aass_F2: 5’- CCTTCAGGTTGAGAACTGGTGTT-3’
Aldh7a1	wt: 510 bp<br>Mut: 323 bp	Aldh7a1_F1: 5’- AGCTCCTCAGGGTAAAGTCC-3’<br>Aldh7a1_rev1: 5’- CTGGTCTTCTTGCTTGTGTTTCC-3’<br>Aldh7a1 wt Rev2: 5’-CCTTGCCTTCTGAAAGTAAGG-3’
**Primers for RNA QC**		
Target gene	Primer sequence	
Aass	Aass ex3for 5’- GCCTGTCTGATTTTGGGAGT-3’<br>Aass ex9 rev 5’- TGAGCATCCTGACGTGTGAG-3’<br>Aass ex2 for 5’- GGCATCACCAAACTGGGCTA-3’	
Aldh7a1	Aldh7a1 ex4 for 5’- CTATTGTCCTGCTAACAATGAGC-3’<br>Aldh7a1 ex10/11 rev 5’- GGCTATGATCTTTGTGACAGCC-3’	

Supplementary Table 3

**Supplementary Table 3**	**Characteristics and Fold Changes (FC[1]) of biomarkers for *Aldh7a1* KO, *Aass* KO and *Aass/Aldh7a1* DKO**											
**Biomarker**	**Feature**			***Aldh7a1* KO**			***Aass* KO**			***Aass/Aldh7a1* DKO**		
	Adduct	***m/z***	***rt***	**plasma**	**brain**	**liver**	**plasma**	**brain**	**liver**	**plasma**	**Brain**	**liver**
Pipecolic acid	M-H	128.0717	0.97	12.5	64.0	6.7	10.1	2.4	12.8	9.8	2.1	9.4
P6C	M+H	128.0706	0.86	↑	↑	9.6	↑	↑	2.0	↑	↑	3.9
6-oxoPIP	M-H	142.0510	2.55	27.2	2.4	23.3	0.3	-4	1.1	2.3	-2	3.5
2-OPP	M+H	186.1125	2.36	256.5	↑	56.0	3.3	ND	5.5	11.0	ND	5.0
Saccharopine	M-H	275.1249	0.60	ND	1.3	2.8	ND	↓	↓	ND	↓	↓
Lysine	M+H	148.1163	0.52	1.1	1.2	1.6	9.4	3.3	3.1	8.1	2.7	2.1
N- Acetyllysine	M+H	189.1233	1.06	1.1	1.3	1.6	2.1	9.7	2.6	3.6	7.4	3.3
Homocitrulline	M-H	188.1041	0.70	0.5	ND	ND	17.7	↑	↑	12.9	↑	↑

Biomarker features were identified in both positive or negative ionization modes as $[M + H]^+$ or $[M - H]^-$ adducts, along with their corresponding mass-to-charge ratio (*m/z*) and retention time (*rt*). Arrows indicate an increase (↑) or decrease (↓) in the intensity of the feature in the genotype sample compared to the wild type (WT). [1]Fold changes (FC) were calculated as the ratio of the mean intensity in knockout (KO) or double knockout (DKO) samples to the mean intensity in WT samples.

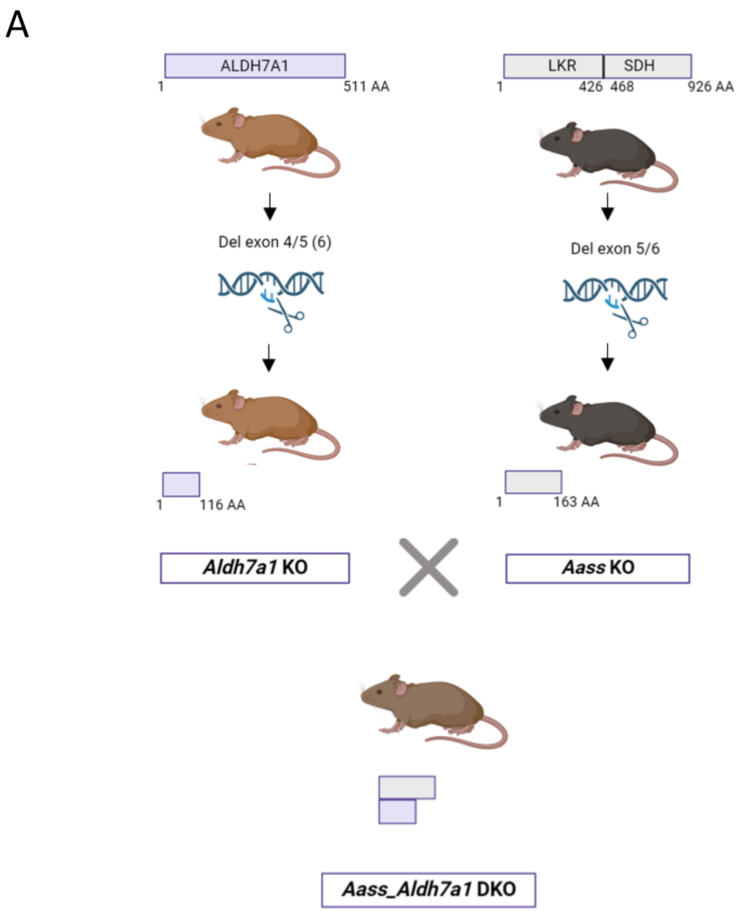

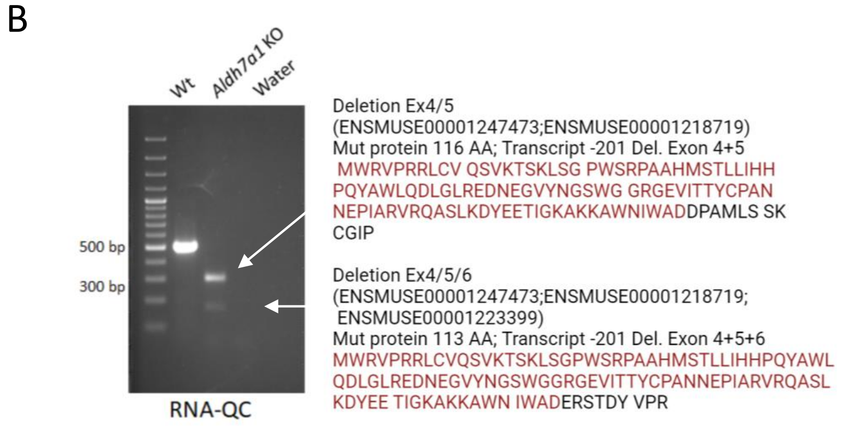

Supplementary Figure 1.

(A) *Overview PDE Mouse Models*. C57BL6/6NCrl *Aldh7a1*$^{\mathrm{em1(IMPC)Hmgu)}}$ mouse model (*Aldh7a1* KO) was generated with CRISPR/Cas technology and crossed to C57BL/6N-*Aass* $^{\mathrm{em1(IMPC)Tcp}}$ (https://www.mousephenotype.org/data/genes/MGI:1353573) (Aass KO) to establish an *Aass/Aldh7a1* DKO mouse model (*Aass_Aldh7a1* DKO). (B) *Validation exon deletion Aldh7a1 KO mouse line.* Aldh7a1 RNA was isolated from kidney tissue of homozygous animals and cDNA from *Aldh7a1* KO animals was used for PCR to detect the deletion of exon 4-5 and in a minor content deletion exon 4-6 (transcript _201). Figure A: Created in BioRender. Coughlin, C. (2025) https://BioRender.com/9vrpv1h. (AA - amino acid; Ex - exon; KO – knock-out; DKO - double KO; QC – quality control)
